# Clinical Reports

**Published:** 1905-12

**Authors:** Herman E. Hayd

**Affiliations:** Buffalo, N. Y; Surgeon to the German Hospital


### CLINICAL REPORTS.

By HERMAN E. HAYD, M. D., F. R. C., ENG., Buffalo, N. Y
Surgeon to the German Hospital.

### FIRST CASE—GUNSHOT WOUND OF THE ABDOMEN—OPERATION—RECOVERY.

An interesting gunshot wound case occurred in the service of Dr. Hayd at the German Hospital. A polish girl, aged fourteen, was brought in, one hour after being shot by a 38 caliber revolver. The ball entered the head of the fourth metacarpal bone, just above the phalangeal joint, of the right hand and came out at the side of the wrist. It then penetrated the abdomen just external to the right rectus, a little below the ribs.

The patient was immediately prepared for operation and anesthesized. The point of penetration through the abdominal parietes was enlarged, simply to be assured that the ball went into the abdominal cavity. A small strip of iodoform gauze was inserted into the wound for superficial drainage. A long incision was made in the median line, between the ensiform cartilage and the umbilicus. The exposed omentum was then lifted out of the wound and the ball was found within its meshes. The transverse colon was brought up into the wound and it was seen that perforation had taken place in its anterior and posterior walls. These perforations were quickly sewed up with fine iodine catgut and reinforced by Lembert's sutures of the same material.

The mesentery, which was considerably lacerated was sewed up and a thorough examination was then made of the small bowels commencing at the angle of Treitz. Each successive portion of bowel was inspected and immediately returned to the peritoneal cavity, much in the same manner as one would haul in a fish line. At no time was there more than four or five inches of bowel exposed to the atmosphere. After thoroughly inspecting the small bowels as far as the ileocecal valve and no perforations being found, the stomach was next examined and a perforation was found in the anterior wall close to the pyloric extremity, which was immediately sewed up. The posterior wall was quickly examined and a perforation found and sewed up. Iodine catgut was used for both perforations, reinforced by Lembert's sutures of the same material. The liver and pancreas being uninjured the abdominal incision was closed by through-and-through silkworm sutures and without drainage.

Another incision was quickly made just above the pubes in the median line and the peritoneal cavity was thoroughly irrigated with a salt solution. Some small pieces of cabbage and potato were washed out which had gravitated into the pelvis; also a small piece of burnt cotton which had evidently been carried into the abdominal cavity from the clothing in the passage of the ball. A glass drainage tube was then inserted into the lower end of the wound which was closed with through-and-through silkworm sutures. The patient then was placed in bed and the body was elevated by placing an iron chair under the head of the bed, the body by this means, being elevated at least ten or twelve inches so as to facilitate drainage. The fluid was withdrawn at short intervals by a long rubber syringe.

The glass tube was left in situ for forty-eight hours. The patient made an uninterrupted recovery. The first three days the temperature ranged from 103 to 101 and then became normal. On the fifth day water and liquid broths were given the patient and gradually a semi-solid diet was ordered.

Advantage was taken in the treatment of this case of the splendid work of Dr. John Young Brown, of St. Louis, who has shown by his report of a great many successful cases of gunshot wounds of the abdomen, that no attention should be given to the point of penetration other than to assure ones self that the ball went through the abdominal parietes. A median incision then always should be made between the ensiform cartilage and the umbilicus, and the bowels, stomach, liver and pancreas can then be thoroughly explored. Another opening should then be made just above the pubes to wash out the peritoneal cavity and to provide for drainage, as Dr. Brown does in every one of his cases. His technic, however, with stab wounds of the abdomen is slightly different, in that he then uses the point of penetration as the guide for his incision because the probabilities are that with a knife or sword stab the bowels would be injured under the point of entrance; but with a gunshot wound such probably would not be the case. However, even with a stab wound it is a good rule to open the abdomen in the median line because the bowels can then be examined from duodenum to sigmoid flexure, by extending the median incision either downwards or laterally.

SECOND CASE—GALLSTONE DISEASE — OPERATION — RECOVERY.

Another case of importance was that of an acute cholecystitis occuring in the seventh week of a typhoid attack. The patient was a young married woman, twenty-five years of age, without children. There was no history of gallstone colic or jaundice, or of any previous attack of typhoid fever. The patient complained of great pain and tenderness and a globular swelling could be felt under the border of the ribs. She was in a very emaciated condition, having a pulse of 136 and a temperature of 103° F, vomiting and suffering great pain.

Operation was immediately decided upon and the patient was prepared and anesthetized. At no time, however, was there complete narcosis. An incision about three inches in length was quickly made, exposing the distended gallbladder. The adhesions about the gallbladder were released and gauze was freely and carefully packed around it. An incision was then made into the gallbladder and nearly a pint of thick, viscid, light-colored fluid, slightly stained with bile, was removed, together with about two hundred gallstones. The gallbladder was sewed to the abdominal wound and a drainage tube inserted. The patient reacted promptly and her convalescence was speedy and uninterrupted.

---

Inflammations of the pleura (*International Journal of Surgery*) are so often of tuberculous origin that it is well to treat all these cases as if they were actually tuberculosis. Hence after operations upon the pleural cavity, an antituberculous plan of treatment should be pursued and the patient kept under observation for some time.

